# Construction, Factor Structure, and Internal Consistency Reliability of the Hospital Physical Therapy Perceived Satisfaction Questionnaire (H-PTPS)

**DOI:** 10.3390/ijerph17165857

**Published:** 2020-08-12

**Authors:** Manuel Albornoz-Cabello, José Manuel Pérez-Mármol, Mª de los Ángeles Cardero-Durán, Cristo Jesús Barrios-Quinta, Luis Espejo-Antúnez

**Affiliations:** 1 Department of Physiotherapy, University of Sevilla, 41009 Sevilla, Spain; malbornoz@us.es; 2 Department of Physiotherapy, University of Granada, 18016 Granada, Spain; 3 Instituto de Investigación Biosanitaria de Granada-ibs.GRANADA, 18012 Granada, Spain; 4 Puente Real II Healthcare Center, 06006 Badajoz, Spain; m.angeles.cardero@gmail.com; 5 Physiotherapy Unit, Andalusian Health Service, 41300 Sevilla, Spain; cristo.barrios@gmail.com; 6 Department of Medical-Surgical Therapy, University of Extremadura, 06006 Badajoz, Spain; luisea@unex.es

**Keywords:** questionnaire, internal consistency, patient satisfaction, patient safety, physical therapy, patient-focused care

## Abstract

Patient satisfaction is a crucial aspect in the evaluation of the quality of health care provided by health services and units, especially in patients that require physical rehabilitation. This study aims to design and analyze the factor structure and internal consistency reliability of the Hospital Physical Therapy Perceived Satisfaction Questionnaire (H-PTPS) measuring the level of physical therapy patient satisfaction in hospital rehabilitation services. This study has a multicenter cross-sectional survey design. This study used the structural validity and internal consistency domains from COSMIN (consensus-based standards for the selection of health status measurement instruments) guideline. The H-PTPS questionnaire consists of 20 closed questions. A sample of 384 adult patients from physical therapy units from three Spanish public hospitals completed this questionnaire. A factor structure and internal consistency reliability analysis were performed. The factor analysis including the 20 items of the H-PTPS showed an adequacy index of 0.922 according to the Kaiser–Meyer–Olkin measure and the Barlett test allowed us to reject the null hypothesis (*p* < 0.001). In the rotated component matrix, four specific factors were obtained, explaining 66.75% of the accumulated variance. All factors present satisfactory internal reliability, achieving Cronbach’s alpha indices and Omega coefficients higher than 0.74. The H-PTPS questionnaire has shown a four-factor solution with satisfactory reliability evaluating the satisfaction of Spanish patients treated in physical therapy units in the hospital rehabilitation services.

## 1. Introduction

Physical therapy patient satisfaction is a relevant construct in the evaluation of the quality of health care rehabilitation services at a clinical, political, and research level [1,2,3]. The complex multidimensionality of the construct has traditionally hindered its definition. In recent years, the theoretical models of quality of care highlight the importance of person-centered care to achieve optimal levels of patient satisfaction. Numerous domains have been identified in patient satisfaction such as the interpersonal component of interventions, and the expectations about health care services after rehabilitation [4,5,6,7]. Several authors have contributed to the paradigm referred to as the expectancy-disconfirmation, explaining the expectation–satisfaction link in the formation of satisfaction judgments of patients who receive physical therapy [8]. Precise information at the different stages of treatment enables patients to have more realistic expectations, increase treatment adherence [8,9], decrease the levels of pain intensity, and have adequate levels of self-efficacy [10]. Hence, patient satisfaction is a crucial aspect in the evaluation of the quality of health care provided by health services and units, especially in patients that require physical rehabilitation. Several approaches have been developed to survey patient perceptions of health care such as suggestion boxes, formal complaints, qualitative methods, or audits. However, satisfaction questionnaires are undoubtedly the most commonly used method [11].

Although the measurement of patient satisfaction is a topic of interest in health systems, Roush et al. [4] indicate that knowing the satisfaction of patients referred to physical therapy units constitutes a complex and difficult task since that there is no a “gold standard” measure in physical therapy for this purpose [4]. This fact could be due to the heterogeneity between physical therapy units at an organizational level, or the type of intervention strategies used and the type of patients treated. This heterogeneity may have a certain influence on the physical therapy patient satisfaction, existing different internal and external factors that may be involved in the patient’s expectations [4]. Several studies have been conducted on the levels of satisfaction including diverse types of pathologies or contexts such as patients with cystic fibrosis [12], physical therapy units from occupational accident insurances [13], or patients receiving outpatient physical therapy [14]. On the other hand, the measurement instruments existing to date on perceived patient satisfaction revealed that some studies failed to report the psychometric properties of the instrument used, and some of these studies reported the patient’s satisfaction based on a single item [15]. This may be explained because of the lack of content attention at the conceptual level of measuring instruments since they should be based on the multidimensional concept of patient satisfaction [11], as well as the difficulty in choosing the most suitable measuring instrument for each specific work environment [4]. For this reason, instruments that measure satisfaction must be designed taking into account the characteristics of the context. A large number of measurement instruments are available for evaluating physical therapy patient satisfaction in several languages such as French [16], Spanish [17], or English [18]. However, to our knowledge, there are still no instruments evaluating patient satisfaction among those attending hospital rehabilitation physical therapy services.

When it comes to designing new measurement tools that evaluate health care quality, it is crucial to understand the factors related to patient satisfaction. It is well known that the patient has been traditionally provided with little therapeutic information. Santos de Almeida et al. [16] reported that healthcare satisfaction must be considered an “emotional” aspect related to the actions of health professionals since their behavior has an impact on the patient’s health. In this line, recent research has shown that precise information at the different stages throughout the treatment enables the user to have more realistic expectations, favoring treatment adherence, and higher levels of satisfaction [8,9]. Additionally, adequate information may be related to lower levels of pain intensity and higher levels of self-efficacy for managing and coping with chronic disease [10]. Nevertheless, physical therapy interventions in the Spanish hospital health care context lack a measurement instrument for evaluating patient satisfaction [17,19]. Hospital-based physical therapy is different from outpatient-based physical therapy in terms of the type of patients assisted (complexity of the disease), the health status of patient (acute/subacute phase) and therapeutic approaches (degree of individualized care), requiring the development of a brand new instrument for the hospital context. To the best of our knowledge, there are no studies that analyze the psychometric properties of a satisfaction questionnaire including multiple items and designed for Spanish patients receiving hospital physical therapy. This instrument could be used by health professionals, managers, and researchers to evaluate the effectiveness and monitor improvements after physical therapy intervention programs.

The main objective of the present study is to design and analyze the factor structure and internal consistency reliability of the Hospital Physical Therapy Perceived Satisfaction Questionnaire (H-PTPS) measuring the level of physical therapy patient satisfaction in hospital rehabilitation services.

## 2. Materials and Methods

### 2.1. Study Design

This study has a multicenter cross-sectional survey design. To design the study we used the structural validity and internal consistency domains from COSMIN (consensus-based standards for the selection of health status measurement instruments) guideline [20,21].

### 2.2. Study Population

**Figure 1:**
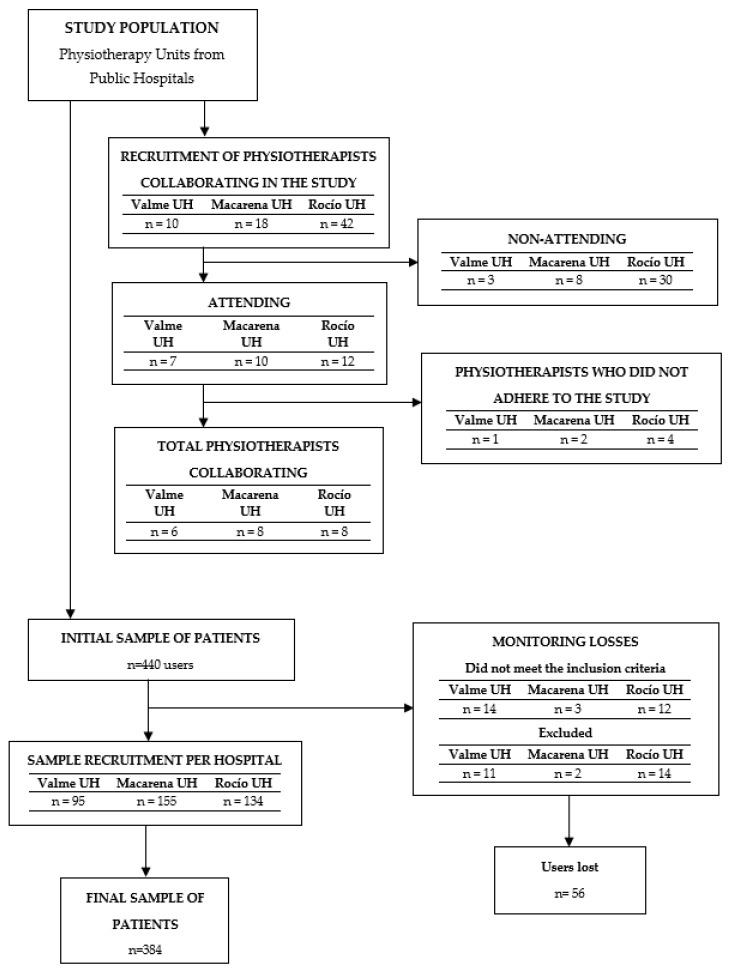
Flow chart of the selection process for the sample.

A total sample of 440 potential participants was approached using systematic random sampling among patients who received physical therapy treatment in the Hospital Physical therapy Units from three public hospitals in Seville, Spain (Virgen de Valme, Virgen de la Macarena and Virgen del Rocio University Hospitals). Since the robustness and accuracy of exploratory factor analysis benefit from large samples and the traditional rules of thumb for inferential statistics advise having a minimum of ten participants per item, we used a sample size higher than 300 participants, determined by Comrey and Lee as a “good” sample [22]. The final sample included 384 patients that met the selection criteria. Figure 1 shows a flow diagram of the selection process for the sample. The study was performed between October 2015 and March 2018.

The inclusion criteria were, (1) being over 18 years old, (2) being able to understand the contents and the aim of the study and questionnaire, and (3) having received treatment in one of the physical therapy units after a referral by a general practitioner in a public hospital from the Spanish National Health System. Those patients who left the physical therapy unit before completing the study and those who were not discharged before the end of this study were excluded.

The research protocol was reviewed and approved by the Institutional Review Board - Research Ethics and Health Research Committee of the Virgen de Valme, Virgen de la Macarena, and Virgen del Rocio University Hospitals (Seville, Spain) with reference: 1584-N-10, 2019-05. All subjects agreed to participate by signing the informed consent form approved by the Review Board. The study protocol was conducted according to the Declaration of Helsinki.

### 2.3. General Procedures

Once the informed consent form was signed, the inclusion and exclusion criteria were applied, and the monitoring losses were quantified. The questionnaire was distributed by assistant researchers that were trained to administer the questionnaire. This action guaranteed the blinding of each patient from the physiotherapists. The questionnaire was distributed after a week of treatment or after the fifth session. The questionnaire administration setting was standardized among all three hospitals. The evaluation was performed in appropriate and separate rooms, as reported by Vanti et al. [19].

### 2.4. The H-PTPS Questionnaire Design Process

The design and elaboration of the measuring instrument were performed in three stages. The first stage consisted of the conceptualization of the H-PTPS questionnaire. Several biomedical databases (PubMed, Cochrane, Scopus, CINAHL, and PEDro) were searched for quantitative and qualitative studies on patient satisfaction. This review was based on levels of patient satisfaction in the health care context across different biomedical fields, as well as focused on measuring instruments evaluating the levels of quality of health care and the user’s satisfaction. The Hospital Physical Therapy Perceived Satisfaction questionnaire (H-PTPS) was based on the content of previous validations published by Beattie et al. [17] and other authors [5,17,19,23,24,25,26]. Moreover, the H-PTPS requested the patient to answer questions specifically relating to his/her experience receiving physical therapy, as indicated by Goldstein et al. [18].

The second stage consisted of the construction of the H-PTPS questionnaire, following the classification model of satisfaction-measuring instruments [27]. The following criteria were established: (1) questions must be multidisciplinary, including aspects of satisfaction perception, while also providing insight about a global issue; (2) the items must be focused on the satisfaction perception concerning the health care process, not only on the satisfaction with the results achieved; (3) the statement must not include any particular issues about a specific pathology, that is, it must be generalizable to any patient; (4) the questions must be inquired through specific facts rather than perceptions. The H-PTPS questionnaire includes questions related to confidence in the staff and the information provided by them, as well as questions regarding the organization of the physical therapy units. The questionnaire was conceptually broken down into three parts: (1) personal and registration data; (2) professional data related to the physical therapy units; (3) the users’ satisfaction data. This questionnaire consisted of 20 closed questions, with simple options on a 5-point Likert scale, ranging from 1 point (very unsatisfied/very bad) to 5 points (very satisfied/very good). 

The third stage was based on the evaluation of the understandability of the questionnaire using a group of experts with extensive experience as physiotherapists (more than ten years). They completed a form to evaluate the understanding of the items from the questionnaire and to offer the possibility of including new items. A pilot study was executed, conducting interviews with patients to explore their perception regarding the positive and negative aspects of hospital physical therapy care as well as the understanding and interpretation of the questions. We ensured that the grade level of the content was appropriate for the general population. This information was used to generate new questions to be included in the questionnaire [11]. This pilot study was conducted from January 2016 to March 2016 at the Virgen de Valme University Hospital. Then, it was statistically analyzed in terms of internal consistency reliability (n = 85), obtaining a good reliability score (Cronbach α = 0.89).

### 2.5. Statistical Analysis

The data analysis was performed using SPSS 20.0. Descriptive statistics were calculated, and the assumption of normality was confirmed for continuous variables. The sex and age-based differences in the mean scores for each of the 20 items were assessed using independent Student *t*. To evaluate the underlying variable structure of the H-PTPS, an exploratory factor analysis of the items was conducted. To reduce the dimensionality of the data set, while retaining as much as possible of the variation present, principal component analysis (PCA) was used [22,28]. The Kaiser–Meyer–Olkin measure (KMO) was used to assess sampling adequacy. The KMO statistic ranges from 0 to 1. A value of 0 indicates that the sum of partial correlations is large relative to the sum of correlations, indicating diffusion in the pattern of correlations. In contrast, a value close to 1 indicates that patterns of correlations are relatively compact. The KMO value should be higher than the acceptable threshold of 0.5 to be satisfactory. Barlett´s sphericity test was used to test for appropriateness. The Barlett’s test for sphericity is also performed to highlight the presence of correlations among the variables. This test is used to evaluate the hypothesis that the correlation matrix is an identity matrix, which indicates that there is no relationship between the items. To evaluate the interactions between items, a Roter Interaction Analysis System was applied [29]. This system allows groups to be grouped into variable-factor sets by applying the Varimax Orthogonal Rotation Model. To analyze the internal consistency, the statistical value of Cronbach´s Alpha and Omega coefficients was calculated. The individual-level reliability was addressed by calculating the standard error of the measure (SEM) for the mean value of each factor, as proposed by Beattie et al. [17]. To calculate the SEM for each of the proposed factors, the following formula was used: SEM = SD √ (1-Cronbach α).

## 3. Results

### 3.1. Sample Description

A total sample of 384 patients receiving physical therapy rehabilitation was recruited. The distribution of the sample in the three hospitals was as follows: University Hospital Valme: 24.7% (*n* = 95), University Hospital Macarena: 40.1% (*n* = 155) and University Hospital Rocío: 35.2% (*n* = 135). Sociodemographic, anthropometric, and clinical characteristics of the sample are presented in Table 1. At baseline, no statistically significant differences were found between males and females for anthropometric/clinical characteristics. The average age distribution was 50.69 ± 14.94 years old. The participants were treated by 22 physiotherapists working in those hospitals (Valme University Hospital: 6; Macarena University Hospital: 8; Rocío University Hospital: 8).

### 3.2. Appropriateness: Sampling Adequacy and Sphericity

The factor analysis of the 20 items showed a Kaiser–Meyer–Olkin measure of sampling adequacy of 0.922. Likewise, the Barlett test allowed us to reject the null hypothesis (sphericity) (*p* < 0.001).

### 3.3. Factor Structure of the H-PTPS Questionnaire

The distribution of variance explained by the factors extracted from the principal component analysis showed a questionnaire structure divided into four factors, explaining 66.75% of the accumulated variance. The first stronger factor explained 44.06% of the variance, followed by a second factor (12.08%); a third factor (5.60%), and a fourth factor (5.02%). In the rotated component matrix, it was observed that each group of items individually and strongly correlated with each component (factor) of the construct (Table 2). A strong relationship was shown between each factor and the rest of the questions (>0.6), except for items Questions 2, 6, and 11. Regarding Factor 1, the question about the patient´s opinion on the treatment schedule (Question 2) and the question about confidentiality and professional secrecy (Question 11) showed correlations that were relatively lower than the rest. Finally, the question about the location of the physical therapy Unit (Question 6) showed a moderate connection (factorial weight = 0.55) with Factor 4.

### 3.4. Internal Consistency Reliability of the H-PTPS Questionnaire

The reliability analysis showed a Cronbach’s alpha and Omega coefficient values >0.75 for all factors of the H-PTPS questionnaire, which is considered good. Although the individual variability (SD) of the total factor scores is slightly high (especially in Factor 2), the Cronbach’s alpha and Omega coefficient value varies from acceptable to good in all of them, demonstrating a better group performance than individual performance. The reliability and the SEM results of each factor of the H-PTPS questionnaire are shown in Table 3.

### 3.5. Descriptive Data for the H-PTPS Questionnaire

The mean values, standard deviation, maximum and minimum of each item in the H-PTPS questionnaire, and grouped by the factor, are shown in Table 3. The evaluator instruction manual and the validated H-PTPS questionnaire in English and Spanish are shown in Table 4 and Table 5.

## 4. Discussion

The main objective of the present study was to design and analyze the factor structure and internal consistency reliability of the Hospital Physical Therapy Perceived Satisfaction Questionnaire (H-PTPS) measuring the level of physical therapy patient satisfaction in hospital rehabilitation services. The factor analysis obtained that the perception of satisfaction with hospital physical therapy services in Spanish patients consisted of four main factors with adequate psychometric properties. This four-factor solution brings together different characteristics of patient satisfaction such as the physiotherapist-patient relationship (Factor 1, Questions 1, 2, 8, 10, 11, 12, and 16), the context in which the treatment is performed in terms of comfort, resources, cleanliness, and privacy (Factor 2, Questions 3, 4, 5, 7, 14, and 15), the physiotherapist-patient communication in terms of received information, tolerance of the opinions, and exploration performed (Factor 3, Questions 17, 18, 19 and 20), and organizational and environment aspects of the health system (Factor 4, Questions 6, 9, and 13). This solution shows a similar structure to the factors obtained by Diógenes et al. [23] who evaluated the dimensionality of satisfaction of older adult Brazilian outpatients receiving physical therapy. These authors conducted the validation study in a network of private clinics from Natal (Brazil), including 385 patients, 64.6% female and 35.4% male. The factors of their questionnaire were termed as patient-physiotherapist interaction, access to and assistance from the medical staff, the physical environment, and general satisfaction. However, Vanti et al. [19,24], in the validation of the Italian version of the Physical Therapy Patient Satisfaction Questionnaire, PTPSQ-I, identified two main factors, including 16 items and 15 items, respectively. These authors included outpatients and hospitalized patients treated with physical therapy, observing that aspects related to treating them with “respect and dignity” contributed as a strong factor to perceived satisfaction. These results seem consistent with the items included in the H-PTPS questionnaire, where Questions 11, 12, 13, 14, and 15 are items about respect and intimacy, being represented in three of the four factors. Further, these aspects seem to be more relevant when there is continuity of care, as usually occurs in physical therapy units [30]. This was justified in the sample recruited for the present study by the number of sessions (days) received by the patients attended in the units where the sample was recruited (25.23 ± 32.58, Table 1). This aspect may have influenced the high internal consistency obtained for the H-PTPS. On the other hand, Beattie et al. [17], in their validation of the Patient Satisfaction with Physical Therapy Care (MedRisk survey), included Spanish speakers from the United States. However, they recruited a sample of outpatients receiving physical therapy. Our measurement instrument was designed for evaluating the satisfaction of patients under physical therapy treatment in the hospital setting. Since they did not include Spanish-speaking people, there are probably differences in cultural, political, and economic characteristics between both populations. The H-PTPS may be used by health professionals, managers, and researchers to evaluate the effectiveness and monitor improvements after physical therapy intervention programs.

Patient satisfaction is usually measured by evaluating the components of healthcare quality. However, it is necessary to differentiate between these components and the actual perception of user satisfaction [29]. Previously, health user satisfaction has been studied in general rehabilitation services [23] and in physical therapy units [13,19,23], but the scales used in these studies did not register aspects such as the satisfaction with other units, included in the fourth factor of H-PTPS questionnaire (Questions 6, 9, and 13) [8,17,19,25]. The fourth factor lets the patient compare the quality of the physical therapy received in the physical therapy units with other health care received by other members of the hospital staff. For this reason, it is necessary to have instruments that measure the satisfaction of the patients from different clinical settings, such as those under hospital physical therapy treatment in rehabilitation services. Recently, some authors have identified factors similar to that included in the H-PTPS, such as Rodríguez Nogueira et al., who included a factor referred to as “environment” with items such as “I observe a lack of coordination between the team of professionals (physiotherapists, doctors, aids, administration staff, etc.) who attend me” [5]. In this sense, potential reasons for the lack of notable improvements in physical therapy patient outcomes and health expenditures could be related to traditional medical care models. Recently, Denninger et al. [26] compared total claims paid and health outcomes obtained in patients with neck and back pain receiving physical therapy treatment via direct access vs. medical referral. These authors concluded that patients’ choice to go to the physiotherapist through direct access may be related to the lower health care expenditures for this type of patient. Hence, the fourth factor could help to detect the impact that these aspects have on the quality of health care.

The individual item contribution to the factorial structure of the instrument is consistent with other studies [5,19,25,26]. More particularly, the most repeated questions in the literature (Questions 8, 9, 16, 17, 18 and 20), which explain most of the variance in the user’s satisfaction construct, belong to the information shared in the medical staff-patient relationship. Specifically, Question 17 of the H-PTPS is included as part of domain two, referred to as “physiotherapist-patient communication”, which is similar to the factor referred to as “capacity of communication” from the study conducted by Rodríguez Nogueira et al. [5] on the validation of a questionnaire focused on the person-centered therapeutic relationships in physiotherapy services. On the other hand, other items included in the H-PTPS have also been reported by other studies, presenting acceptable psychometric properties. For example, the item “respect received by the physiotherapist during assistance” (Question 12) has also been reported by Mangset et al. [25]. Item Question 6 of this questionnaire referred to as “orientation, location of the physical therapy unit” included in Factor 4 is similar to that reported by the study by Beattie et al. [17], which included “the office location is convenient”. Finally, the items “how difficult was it for him or her to find the physical therapy unit?” (Question 6), “what is his or her opinion about the care schedule you were offered?” (Question 2) and “to what extent does he or she think that confidentiality or professional secrecy has been respected?” (Question 11) have been reflected in the study by Vanti et al. [19]. Those similarities could explain the positive association between communication, the correct transmission of information, and the user’s satisfaction [31]. Ruben et al. [10] pointed out that positive perceptions of communication provided to the patient were related to higher levels of patient self-efficacy, which in turn was related to lower levels of pain intensity and pain interference. Nevertheless, Dwamena et al. [32] in a Cochrane review concluded that the impact of interventions, with a focus on the patient and not only on their health problems, has not so far been adequately evaluated in satisfactory terms. Therefore, the psychometric results of the questionnaire proposed in the present study could provide a valid and reliable communicative tool to analyze the aspects that may influence the perceived satisfaction among patients under physical therapy treatment.

In terms of reliability, the H-PTPS questionnaire showed high internal consistency with Cronbach alphas and Omega coefficients greater than 0.7 in all factors. These results are in line with previous studies conducted in the field of physical therapy with similar populations in terms of sample size, the predominance of female patients, or average age [13,17,18,19,23]. The Diógenes et al. study [23] reported reliability indices exceeding the values proposed for exploratory studies, with a Cronbach’s alpha coefficient of 0.943. These authors also highlight the physical therapist-patient interaction as the key aspect involved in the level of satisfaction with the care received, but they referred to older adult patients. The lack of communication and/or information between the medical staff and the patients has been reported as a relevant point in the loss of confidence in healthcare and, therefore, in the patient’s satisfaction [33,34,35]. The identification of this construct in the perceived satisfaction with the quality of the health services received (Factor 3; Cronbach alpha = 0.889), is also consistent with recent studies that have reviewed the modalities in which the clinical information is transmitted in the clinical discharge report and its influence on the satisfaction and understanding of the information by the patient. These studies also reported the preferences of patients in the transmission of that information. Their results showed the need of improving the communicative process in the transmission of clinical information at discharge to promote patient satisfaction [36,37].

The low values of SEM, mainly observed in Factors 1 and 3, indicate a low degree of error, as observed in Beattie et al. [17]. The variability observed in the items associated with Factors 2 (Questions 3, 4, 5, 6, 7 and 15) and 4 (Questions 9 and 13) in comparison to other studies may be explained by the content validity process followed in the present study. Furthermore, the factor analysis used explores the nature of scales and the interrelation among elements, but these aspects depend on the empirical data available [38]. The same reason could justify the existence of different models explaining the same construct (patients’ satisfaction under physical therapy rehabilitation), such as the PTPSQ-I [24] or the Med Risk Instrument for Measuring Patient Satisfaction with Physical Therapy Care [13]. These validation studies have been conducted in countries other than Spain, such as the United Kingdom [39], United States [40], or Japan [41], in both the public and private sectors. However, there are a limited number of studies in the Spanish language. Taking into account the results obtained and that satisfied patients are more likely to adhere to the physical therapy interventions and have a higher health-related quality of life [31], we suggest for future studies to implement the proposed H-PTPS questionnaire to compare how it could influence the perception of users of physical therapy in their adherence to physical therapy treatment between levels of care (primary care vs. hospital care) or between those who pay for physical therapy care and those who do not [19].

The information obtained by the H-PTPS administration could promote clinical practices for improving the quality of health services. This questionnaire could be recommended for the evaluation of the user’s satisfaction in physiotherapy units at the hospital setting for Spanish and similar populations. However, some limitations deserve to be mentioned. Firstly, the lack of control over the evolution of the participants’ disease at the time they were included in the study sample could produce a bias. Therefore, it seems relevant that the general satisfaction is independent of the clinical result and then of disconfirmation of their initial expectations [8]. However, including patients with diverse pathologies has probably enhanced the generalizability of the results. Secondly, although this research has a multicenter design, it was only conducted in hospital rehabilitation services from three Spanish public hospitals. Thus, the H-PTPS scale probably cannot be generalized to other health care settings. Thirdly, given the limited studies that retain items from other well-designed satisfaction questionnaires with good psychometric properties [4], a confirmatory analysis design should be conducted in future studies to cross-validate the factor structure. However, this exploratory factor analysis is appropriate for exploring the nature of scales and item interrelationships [22]. Fourthly, the analyses of the present study were focused on factor structure and internal consistency reliability of the H-PTPS. However, according to the COSMIN guideline, other psychometric properties should be studied in future research, such as reliability (test-retest, inter-rater), criterion validity (concurrent validity, predictive validity), responsiveness, and interpretability. Finally, an additional limitation may be the skew in the individual items, suggesting a ceiling effect.

## 5. Conclusions

The findings of this study allow health professionals to have a new reliable measurement tool to measure the degree of satisfaction perceived by patients receiving physical therapy treatment in a hospital environment. This knowledge could allow the implementation of future practices aimed at improving indicators related to satisfaction. These indicators would allow an increase in the quality of the service given in the hospital physical therapy units. Moreover, the quality of the service could be compared with other medical units or departments.

The evaluation of patients’ satisfaction is a high priority for many healthcare providers. Our findings are in line with the relevance of having a measurement instrument that is simple to administer and allows one to analyze satisfaction with the healthcare process. Satisfaction with the healthcare provided encompasses complex multidimensionality, requiring instruments that validly and reliably measure the degree of satisfaction. The design of evaluation models that show factors that are predictive of adherence and user perception regarding the treatment received has been documented in some countries. However, in Spain, the number of studies is limited.

## Figures and Tables

**Table 1:** Demographic, anthropometric and clinical characteristics of the sample.

Characteristics	Mean (SD)/*n* (%)	Median	Minimum	Maximum
Age	50.69 (14.94)	51	19	88
Weight (kg)	74.65 (15.17)	72	45	130
Height (cm)	164.77 (10.36)	165	99	191
Body mass index (kg/m^2^)	27.36 (5.08)	26.57	17.17	47.94
Days in the waiting list	30.07 (56.65)	15	0	730
Time spent in the unit (minutes)	69.97 (33.82)	60	5	190
Number of sessions (days)	25.23 (32.58)	15	0	300
**Gender**				
Male	136 (35.4%)			
Female	248 (64.6%)			
**Distribution of the sample**				
Valme UH	95 (24.7%)			
Rocío UH	135 (35.2%)			
Macarena UH	155 (40.1%)			

SD = Standard Deviation, Kg = Kilograms, cm = centimeters, m = meters, UH = University Hospital.

**Table 2:** Rotated component matrix *.

	Component (Factors)			
	1	2	3	4
Satisfaction with Physical therapy care (Q1)	**0.81**	0.21	0.13	0.13
Care schedule offered (Q2)	**0.48**	0.31	0.00	0.37
The comfort of the waiting room (seats, lighting, noise, ventilation) (Q3)	0.09	**0.84**	0.08	0.15
The comfort of the treatment room (seats, lighting, noise, ventilation) (Q4)	0.18	**0.82**	0.14	0.08
Valuation of material resources, treatment room (Q5)	0.09	**0.81**	0.05	0.22
Orientation, location of the Physical therapy Unit (Q6)	0.25	0.33	0.13	**0.55**
Cleanliness of the Physical therapy Unit (Q7)	0.24	**0.61**	0.14	0.31
Valuation of the information provided by the physiotherapist (Q8)	**0.64**	0.16	0.32	0.33
Valuation of the information provided by other medical staff (doctors, nurses, and administration) (Q9)	0.20	0.29	0.14	**0.78**
Confidence in the care provided by the physiotherapist (Q10)	**0.84**	0.09	0.19	0.09
Respect for confidentiality and professional secrecy (Q11)	**0.46**	0.25	0.22	0.39
Respect received by the physiotherapist during assistance (Q12)	**0.73**	−0.00	0.28	0.27
Respect received by other members of the staff (doctors, nurses, and administration) during assistance (Q13)	0.19	0.07	0.33	**0.79**
Valuation of the degree of privacy during the physical therapy treatment (Q14)	0.09	**0.63**	0.43	0.13
Sharing the same room with other patients during treatment (Q15)	0.03	**0.72**	0.36	0.09
Valuation of the attention given by the physiotherapist to the patient (Q16)	**0.74**	0.07	0.37	0.09
Information received about the treatment performed (Q17)	0.43	0.20	**0.67**	0.24
Tolerance on the opinion about the treatment received (Q18)	0.20	0.23	**0.70**	0.26
Valuation of the exploration performed by the physiotherapist before the treatment (Q19)	0.48	0.22	**0.68**	0.06
Information received about exploration before the treatment (Q20)	0.35	0.26	**0.74**	0.22

* Extraction method: Principal component analysis. Rotation method: Varimax normalization with Kaiser; Bold: these factorial weight are put in bold to highlight which were the items what correspond to each factor.

**Table 3:** Mean values, standard deviation, minimum and maximum of each item of the survey and metric properties of the four factors.

	Min	Max	Mean	SD	α	ω	SEM
**Factor 1**			**4.51**	**0.761**	**0.87**	**0.85**	**0.27**
Satisfaction with Physical therapy care (Q1)	1	5	4.58	0.709			
Care schedule offered (Q2)	1	5	4.05	1.033			
Valuation of the information provided by the physiotherapist (Q8)	1	5	4.56	0.739			
Confidence in the care provided by the physiotherapist (Q10)	1	5	4.64	0.687			
Respect for confidentiality and professional secrecy (Q11)	1	5	4.42	0.772			
Respect received by the physiotherapist during assistance (Q12)	1	5	4.74	0.656			
Valuation of the attention given by the physiotherapist to the patient (Q16)	1	5	4.59	0.731			
**Factor 2**			**3.74**	**1.203**	**0.78**	**0.88**	**0.56**
The comfort of the waiting room (seats, lighting, noise, ventilation) (Q3)	1	5	3.44	1.083			
The comfort of the treatment room (seats, lighting, noise, ventilation) (Q4)	1	5	3.69	1.025			
Valuation of material resources, treatment room (Q5)	1	5	3.70	1.000			
Cleanliness of the Physical therapy Unit (Q7)	1	5	4.11	.893			
Valuation of the degree of privacy during the physical therapy treatment (Q14)	1	5	3.94	1.192			
Sharing the same room with other patients during treatment (Q15)	1	5	3.59	2.028			
**Factor 3**			**4.24**	**0.891**	**0.89**	**0.79**	**0.30**
Information received about the treatment performed (Q17)	1	5	4.27	0.873			
Tolerance of the opinion about the treatment received (Q18)	1	5	4.06	1.018			
Valuation of the exploration performed by the physiotherapist before the treatment (Q19)	1	5	4.31	0.850			
Information received about exploration prior to the treatment (Q20)	1	5	4.33	0.826			
**Factor 4**			**4.16**	**0.89**	**0.75**	**0.75**	**0.45**
Orientation, location of the Physical therapy Unit (Q6)	1	5	4.01	0.970			
Valuation of the information provided by other medical staff (doctors, nurses, and administration) (Q9)	1	5	4.13	0.872			
Respect received by other members of the staff during assistance (Q13)	1	5	4.34	0.830			

Min = Minimum, Max = Maximum, SD = Standard Deviation, α = Cronbach alpha, ω = Omega coefficient, SEM = Standard error of the measure.

**Table 4:** Evaluator manual and the items of the English translation of the Hospital Physical therapy Perceived Satisfaction Questionnaire.

Hospital Physical Therapy Perceived Satisfaction Questionnaire—H-PTPS					
Answer the following questions according to your satisfaction with the Physical Therapy assistance received at the hospital where you have received treatment, using the following scale: 1—Very unsatisfied/Very bad 2—Unsatisfied/Bad 3—Neither satisfied nor unsatisfied/Neither good nor bad 4—Satisfied/Good 5—Very satisfied/Very Good					
1. How satisfied are you with the physical therapy care received in this Unit?	1	2	3	4	5
2. What is your opinion about the care schedule you were offered?	1	2	3	4	5
3. How do you feel about the comfort (seats, lighting, noise, ventilation) of the waiting room?	1	2	3	4	5
4. How do you feel about the comfort (seats, lighting, noise, ventilation) of the treatment room?	1	2	3	4	5
5. How do you value the material of the treatment room (stretchers, equipment, pulley systems)?	1	2	3	4	5
6. How difficult was it for you to find the Physical therapy Unit?	1	2	3	4	5
7. What do you think about the cleanliness of the Physical therapy Unit?	1	2	3	4	5
8. How well did you understand the information given by the physiotherapist?	1	2	3	4	5
9. How well did you understand the information given by other members of the staff (doctors, administration, nurses, nursing assistants, wardens, porters/watchmen)?	1	2	3	4	5
10. What level of confidence do you have in the assistance given by the physiotherapist?	1	2	3	4	5
11. To what extent do you think that confidentiality or professional secrecy has been respected?	1	2	3	4	5
12. How do you value the respect shown by the physiotherapist?	1	2	3	4	5
13. How do you value the respect shown by other members of the staff (doctors, administration, nurses, nursing assistants, wardens, porters/watchmen)?	1	2	3	4	5
14. What do you think about privacy during the treatment performed by the physiotherapist?	1	2	3	4	5
15. How do you value the fact of sharing the same room with other patients while you are receiving an individual treatment of physiotherapy?	1	2	3	4	5
16. How do you value the attention paid to you by the physiotherapist?	1	2	3	4	5
17. How do you consider the information you were given about the physical therapy treatment you received?	1	2	3	4	5
18. To what extent were you allowed to give your opinion about the treatment you received?	1	2	3	4	5
19. How do you value the tests or explorations before the treatment, performed by the physiotherapist?	1	2	3	4	5
20. How do you consider the information provided about the tests or explorations performed by the physiotherapist?	1	2	3	4	5

**Table 5:** Evaluator manual and the items of the Spanish validated Hospital Physical therapy Perceived Satisfaction Questionnaire.

Cuestionario de Satisfacción Percibida de Fisioterapia Hospitalaria					
Conteste a las siguientes preguntas según su satisfacción con la asistencia de Fisioterapia recibida en el Hospital donde ha recibido tratamiento, utilizando la siguiente escala: 1—Muy insatisfecho/Muy mal 2—Insatisfecho/Mal 3—Ni satisfecho ni insatisfecho/Ni bien ni mal 4—Satisfecho/Bien 5—Muy satisfecho/Muy bien					
1. ¿Cómo se encuentra de satisfecho con la atención fisioterapéutica recibida en esta unidad?	1	2	3	4	5
2. ¿Qué opinión tiene sobre el horario de asistencia que le ofrecieron?	1	2	3	4	5
3. ¿Cómo valora la comodidad (asientos, luces, ruidos, ventilación) de la sala de espera?	1	2	3	4	5
4. ¿Cómo valora la comodidad (asientos, luces, ruidos, ventilación) de la sala de tratamiento?	1	2	3	4	5
5. ¿Cómo valora el material de la sala de tratamiento (camillas, aparatos, sistemas de poleas)?	1	2	3	4	5
6. ¿Cómo le resultó orientarse para localizar esta unidad de Fisioterapia?	1	2	3	4	5
7. ¿Cómo le resultó la limpieza de la unidad de fisioterapia?	1	2	3	4	5
8. ¿Cómo entendió la información que le daba el/la fisioterapeuta?	1	2	3	4	5
9. ¿Cómo entendió la información que le daban otros/as profesionales (médicos, administrativos, enfermeros, auxiliares de enfermería, celadores, bedeles)?	1	2	3	4	5
10. ¿Qué grado de confianza tiene en la asistencia que le ha prestado el/la fisioterapeuta?	1	2	3	4	5
11. ¿Cómo cree que se ha respetado la confidencialidad o el secreto profesional de los datos clínicos?	1	2	3	4	5
12. ¿Cómo valora el respeto con el que le ha tratado el/la fisioterapeuta?	1	2	3	4	5
13. ¿Cómo valora el respeto con el que le han tratado los otros/as profesionales (médicos, administrativos, enfermeros, auxiliares de enfermería, celadores, bedeles)?	1	2	3	4	5
14. ¿Cómo valora el grado de intimidad mientras el/la fisioterapeuta le realizaba el tratamiento?	1	2	3	4	5
15. ¿Cómo valora el hecho de compartir la misma habitación con otros pacientes mientras usted está recibiendo un tratamiento individual de fisioterapia?	1	2	3	4	5
16. ¿Cómo valora la disposición del fisioterapeuta para escucharle cuando lo ha necesitado?	1	2	3	4	5
17. ¿Cómo considera la información que le dieron sobre el tratamiento fisioterapéutico que le realizaron?	1	2	3	4	5
18. ¿Hasta qué punto le permitieron dar su opinión sobre el tratamiento que le realizaron?	1	2	3	4	5
19. ¿Cómo valora las pruebas o exploraciones previas al tratamiento, realizadas por parte del fisioterapeuta?	1	2	3	4	5
20. ¿Cómo considera la información que le dieron sobre las pruebas o exploraciones realizadas por parte del fisioterapeuta?	1	2	3	4	5
